# Habitat suitability and protected area coverage for an expanding cougar *Puma concolor* population in Canada

**DOI:** 10.1002/ece3.70228

**Published:** 2024-08-30

**Authors:** Jennifer A. Christoff, Eleanor S. Devenish‐Nelson

**Affiliations:** ^1^ Department of Biomedical Sciences, College of Medicine and Veterinary Medicine University of Edinburgh Edinburgh UK

**Keywords:** connectivity, habitat suitability model, human–carnivore coexistence, protected areas, *Puma concolor*, species distribution model

## Abstract

Successful conservation of expanding large carnivore populations and management of human–wildlife conflict to promote coexistence requires sufficient spatiotemporal knowledge to inform appropriate action. In Canada, cougars (*Puma concolor*) are expanding their range eastwards and little research is available for use in decision making by land managers and conservation planners. To inform proactive management regarding expanding populations of cougars in Canada, we utilized open‐source cougar presence and land‐cover data in a maximum entropy habitat suitability model to determine potentially suitable habitat for cougars across the country. We then used a gap analysis to determine the effectiveness of existing formal protected areas to protect potential cougar habitat. Suitable habitat exists for range‐expanding cougars dispersing eastwards through the central and eastern provinces to the Atlantic coast. While the habitat is highly fragmented, the highest suitability occurs in areas of medium road density, indicating that the potential for new human–cougar conflict will likely involve residents of exurban and rural areas. Protected areas offered 16% coverage of suitable habitat, although most protected areas that overlap predicted cougar habitat are not large enough to effectively conserve the large home range requirements of cougars. Synthesis and Applications: High fragmentation of suitable habitat and the potential for human–wildlife conflict requires proactive management to ensure appropriately sized and connected areas are maintained for the establishment of expanding cougar populations. Many of the management actions intended to aid in the conservation of cougars and their habitat can also serve to mitigate potential human–cougar conflict arising as a consequence of an expanding population, such as highway wildlife crossing structures and formal habitat protection.

## Graphical abstract

To inform proactive management of expanding populations of cougars in Canada, we utilized open‐source cougar presence and land‐cover data in a maximum entropy habitat suitability model to determine potentially suitable habitat for cougars across the country. High fragmentation of suitable habitat and the potential for human–wildlife conflict requires that appropriately sized and connected protected areas are maintained for the establishment of expanding cougar populations.

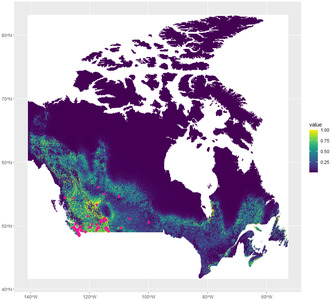

## 1 INTRODUCTION

Globally, large carnivore populations are under threat of extinction (Hoeks et al., 2020), with 64% classified as threatened and 80% undergoing population declines (Wolf & Ripple, 2018). These population changes are largely due to habitat loss and fragmentation (Wolf & Ripple, 2018), human–wildlife conflict (Woodroffe, 2000), overexploitation, and declines in prey species (Wolf & Ripple, 2018). Many species have been lost from portions of their historic ranges, leading to the reduction or loss of ecosystem functioning (Hoeks et al., 2020), as well as the loss of social value that can come from coexisting with charismatic species on the landscape (Rode et al., 2021). While re‐introducing large carnivores is fraught with social and ecological challenges (Mbuh & Vruno, 2018; Rode et al., 2021), some species, such as gray wolves (*Canis lupus*) and brown bears (*Ursus arctus*) have successfully expanded naturally into portions of their historic ranges (Bijl & Csányi, 2022; Jerina & Adamič, 2008). Facilitating passive expansion into extant ranges through active conservation and management potentially restores ecological function and reduces extinction risk (Wolf & Ripple, 2018) but can increase the risk of human–wildlife conflict when large carnivores return to areas where they were long absent (Rode et al., 2021).

Understanding the factors that define species distributions allows for effective conservation planning for protected area establishment, prioritization of critical habitat, and identification of movement corridors and future habitat landscapes (McShea, 2014), particularly in areas of habitat connectivity through areas of increasing anthropogenic infrastructure (Pratzer et al., 2022) and for species that are expanding into historic ranges (LaRue & Nielsen, 2008). Habitat suitability models (HSMs) can be used as an adaptive management tool that provides conservationists, decision‐makers, and other stakeholders a visual presentation of current and potential future species distributions and a starting point for informed, and potentially proactive, planning and policy (Gantchoff, Conlee, Boudreau, et al., 2022; McShea, 2014). This becomes particularly important for large carnivores (Gantchoff, Conlee, Boudreau, et al., 2022) given potential trophic cascades (LaRue & Nielsen, 2015; Wolf & Ripple, 2018) and increased human–wildlife conflict when they use anthropogenically‐modified landscapes as habitat (Buderman et al., 2018; Pratzer et al., 2022).

Decreasing forest cover and increasing human population and road densities have negative impacts on the survival of large carnivores (Cimatti et al., 2021; McShea, 2014). Roads are a primary concern for maintaining large carnivore populations, with numerous negative direct and indirect effects such as, for example, direct mortality and reducing habitat connectivity by fragmenting the landscape (Wolf & Ripple, 2018). While the ability of these species to safely disperse or expand their range within or through human‐dominated areas is constrained more by anthropogenic pressures than by available suitable habitat (Pratzer et al., 2022), the heterogeneity of human‐inhabited areas and enforcement of policies favorable to large carnivores can contribute to their successful expansion (Cimatti et al., 2021; Wolf & Ripple, 2018). Due to the long‐distance movement and large home ranges of these species, habitat connectivity is necessary to allow for species expansion and reestablishment, with Protected Areas (PAs) and Other effective area‐based conservation measures (OECMs), even those relatively small in area, playing an essential role to ensure large carnivore survival (Wolf & Ripple, 2018).

Prior to the 1900s, the cougar (*Puma concolor*, also commonly known as mountain lion or puma, and katalgar in Cree) ranged over much of the continental Americas. Their extant range now encompasses most of South and Central America, the west coast of North America, and isolated patches in the mid‐west (Nielsen et al., 2016). The Canadian cougar range historically extended as far eastwards as the province of Nova Scotia (Government of Canada, 2021; Hood & Neufeld, 2004; Lemelin, 2008). Due to hunting and persecution (Lemelin, 2008; Rosatte, 2011), it is believed that the original eastern populations of cougars were extirpated by the 1940s (Mallory et al., 2012; Rosatte, 2011) leaving an extant Canadian range extending from northern British Columbia and the west coast to approximately the western edge of Saskatchewan in the east (Nielsen et al., 2016). Within the past few decades, there have been numerous documented sightings and DNA evidence from sites within Canada and the USA many hundreds of kilometers beyond the eastern edge of this range (Jenks, 2018; Lang et al., 2013; Mallory et al., 2012; Rosatte, 2011; Rosatte et al., 2015) prompting the argument that cougars are expanding their range eastwards. The current presumption is that these occurrences have been individuals from western populations (LaRue & Nielsen, 2008, 2015) or occasional escaped or released pets (Lemelin, 2008; Rosatte et al., 2015), and not from an isolated remaining eastern population. To successfully manage any such expansion, it is necessary to understand cougar's use of the landscape, responses to habitat disturbance (Avila‐Najera et al., 2017) and anthropogenic features (Dickie et al., 2020), as well as local population ecology (Dellinger et al., 2018).

To fill some of these information gaps, our aims were to (1) determine suitable geographic areas for an expanding cougar population in Canada and (2) evaluate the coverage of current protected areas for suitable habitat to better understand the conservation implications across the landscape for this population. Currently, insofar as we are aware, no published HSM for cougars covering the whole of Canada exists. Cougars are listed as Data Deficient for the eastern subpopulation (*Puma concolor couguar*) in Canada (Government of Canada, 2021); therefore, such information could be used to inform conservation and protected area management decisions (Adams et al., 2015; Gantchoff et al., 2021; Karelus et al., 2021; Swan et al., 2021) and also provide insight into potential areas of high human–cougar conflict (Gantchoff et al., 2021; Smereka et al., 2020; Teixeira et al., 2020).

## 2 METHODS

### 2.1 Producing the habitat suitability model

#### 2.1.1 Study area

**FIGURE A1:**
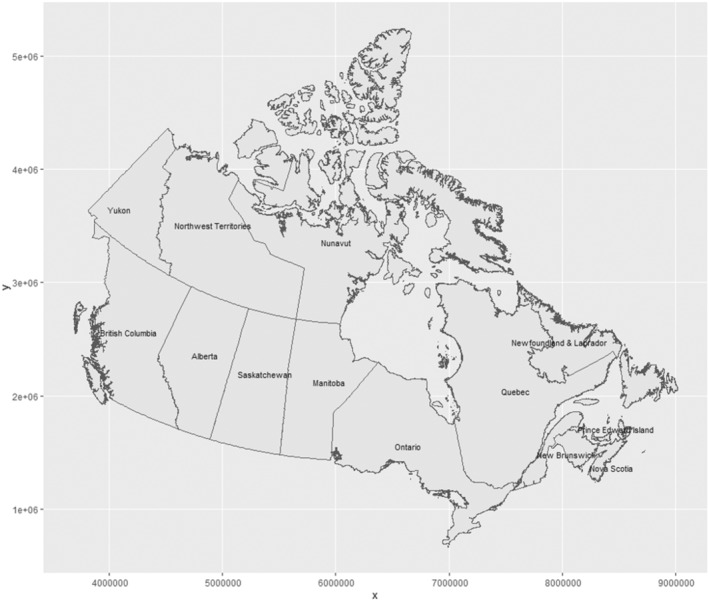
Map of Canada showing individual provinces.

The study area comprises the whole of the country of Canada (Figure A1). Covering a total 9,984,670 km^2^, with over 27% being north of the treeline, and a population of 34.5 million people (Statistics Canada, 2012), Canada has a population density of 3.5 people per km^2^. The country is divided into 10 provinces and three Northern territories.

#### 2.1.2 Model data

Cougar occurrence data (Christoff, 2022) was imported directly into R version 4.1.1 (R Core Team, 2021) from the Global Biodiversity Information Facility (Derived dataset GBIF.org, 2022) using the ‘rgbif’ package (Chamberlain et al., 2021) and the GBIF taxon key for ‘*Puma concolor*’. We specified all Canadian occurrences up to December 2021 that had a ‘human observation’ or ‘material sample’ basis of record and GPS coordinates with no geospatial issues. Points were visually inspected and any duplicate records were discarded.

Environmental parameters were chosen based on those that have consistently been found to be significant in the literature for models of habitat suitability and resource selection functions for cougars in the United States (Buderman et al., 2018; Dickson et al., 2013; Mbuh & Vruno, 2018) and small areas of Alberta and Saskatchewan (Morrison et al., 2014; Smereka et al., 2020). The parameters included elevation (Amatulli et al., 2018), forest cover, water and wetlands, urbanization (Tuanmu & Jetz, 2014), and road density (Meijer et al., 2018) at a 1 km resolution (Table 1). While prey availability has been identified as important for determining cougar habitat for some cougar populations (Buderman et al., 2018), given that white‐tailed deer (*Odocoileus virginianus*) are widespread across the southern half of Canada, with more specific abundance data unavailable, prey availability was not incorporated. Given prior observations that cougars are equally resident in equatorial and high‐latitude climates, climate variables have not been included in many cougar habitat models (Buderman et al., 2018; Dickson et al., 2013; Gantchoff et al., 2021; Mbuh & Vruno, 2018; Smereka et al., 2020) and, therefore, were not used here. Habitat data were downloaded, imported into R, and masked to the land extent of Canada using the ‘rgdal’ package (Bivand et al., 2021). The reduced version of the EarthEnv Consensus Land Cover data was used as the full version incorporates older sub‐pixel imagery (Tuanmu & Jetz, 2014) and the resolution of sub‐pixel data is less important given the large home ranges of cougars. The Global Roads Inventory Project (GRIP) road vector layer (Meijer et al., 2018) was used to create a 1 km road density layer using kernel density in ArcGIS Pro 3.2.0, which is also a proxy for urban areas and anthropogenic disturbance. To limit multi‐collinearity of continuous habitat variables, we retained variables with a Pearson correlation coefficient of |*r*| < .7 (Dormann et al., 2013).

**TABLE 1:** Variables and data sources used in the habitat suitability models.

Model parameter	Data source	Description
Elevation	EarthEnv—Topography 2018	Based on 250 m GMTED2010 and 90 m SRTM4.1dev at a 1 km spatial grain
Forest cover	EarthEnv—Consensus land cover 2014	Contains 12 data layers integrating GlobCover (v2.2), MODIS (v051), and GLC2000 (v1.1) at a spatial resolution of 1 km
Water and wetlands	EarthEnv—Consensus land cover 2014	
Urbanization	EarthEnv—Consensus land cover 2014	
Road density	GRIP Global Roads Database 2018	1 km road density created from GRIP4 vector dataset using kernel density in ArcGIS Pro 3.2.0
Cougar occurrences	GBIF	Open‐access, standardized database of species records

#### 2.1.3 Habitat suitability model

Habitat suitability modeling was conducted using a maximum entropy (Maxent; Phillips et al., 2021) model, as this model type has consistently outperformed other models in terms of predictive power (Duan et al., 2014; Wisz et al., 2008) and has greater stability from the effects of differential sensitivity to variables (Duan et al., 2014) and small sample sizes (Duan et al., 2014; Merow et al., 2013; Wisz et al., 2008). Modeling was performed using the ‘dismo’ R package (Hijmans et al., 2021) that implements the Maxent software (version 3.4.3, Phillips et al., 2021). Specifically, 5000 random background points (pseudo‐absences) were generated through the ‘dismo’ package (Hijmans et al., 2021) by the Maxent software (Phillips et al., 2021) and limited to terrestrial areas by masking of open water (Elith et al., 2010). We visually ensured that these did not overlap presences. As the sampling effort was unknown and Maxent's regularization method is reliable (Elith et al., 2010), the default settings of this software were used. The model was subsequently trained with 80% of the occurrence and absence points and tested with the remaining 20% using five‐fold cross‐validation. As cross‐validation showed the model to be consistent and a good fit, a final model was created using all data points to increase the size and resultant accuracy of the modeled dataset (Wisz et al., 2008).

Models were evaluated using the Area Under the Receiver Operator Curve (AUC) because of its reliability as an indicator of predictive accuracy for presence‐only models (Merow et al., 2013). AUC values over 0.9 indicate an excellent result (Duan et al., 2014). The Maxent jackknife argument (Phillips et al., 2021) was used to determine the response function and permutation importance of each predictor variable. We transformed the predicted suitable habitat into a binary suitability map using a threshold probability set to a sensitivity of 90%. A threshold is required to produce the visually practical binary map from a predicted occurrence map and, with limited threshold selection methods available for presence‐only data (Liu et al., 2013), the fixed sensitivity method was used. A sensitivity, rather than specificity, threshold was chosen because the randomly assigned background ‘absences’ are not necessarily true absences (Merow et al., 2013) and it was more important to correctly predict true presences for the purposes of this model.

### 2.2 etermination of landscape implications for suitable habitat

To evaluate the effectiveness of current protected areas and OECMs for protecting potential cougar habitat from anthropogenic disturbance and land use change, the proportion of suitable habitat under existing protected areas and OECMs was determined using the ‘terra’ R package (Hijmans et al., 2023). Using the binary map, potential cougar habitat was extracted to calculate the percentage under formal protection overall and in protected areas larger than 300 km^2^. Protected area and OECM shapefiles were downloaded from the World Database on Protected Areas (UNEP‐WCMC & IUCN, 2022).

## 3 RESULTS

**FIGURE 1:**
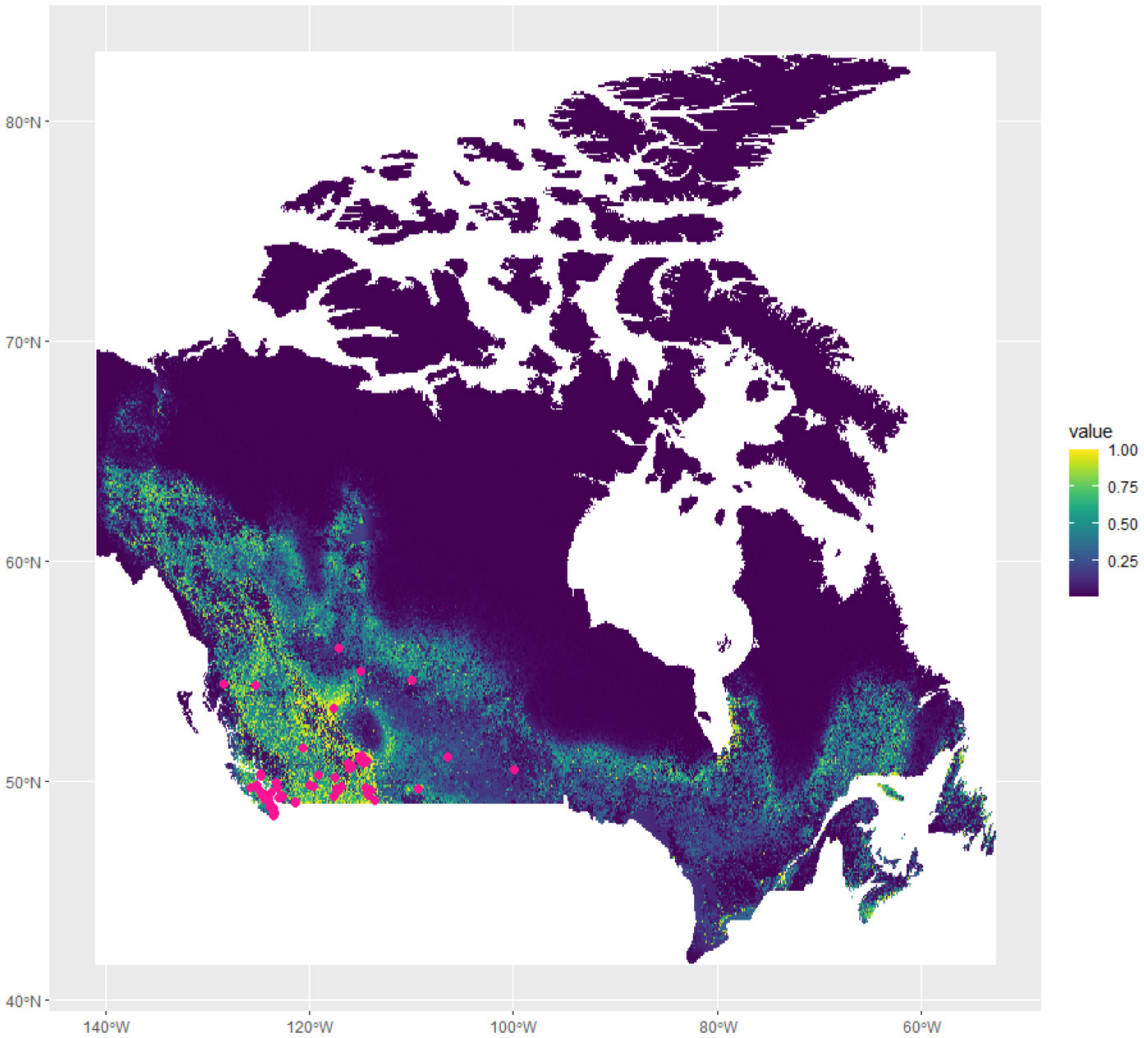
Predicted habitat suitability map for cougar (*Puma concolor*) in Canada showing recorded presence points used to inform the Maxent model created in R.

**FIGURE 2:**
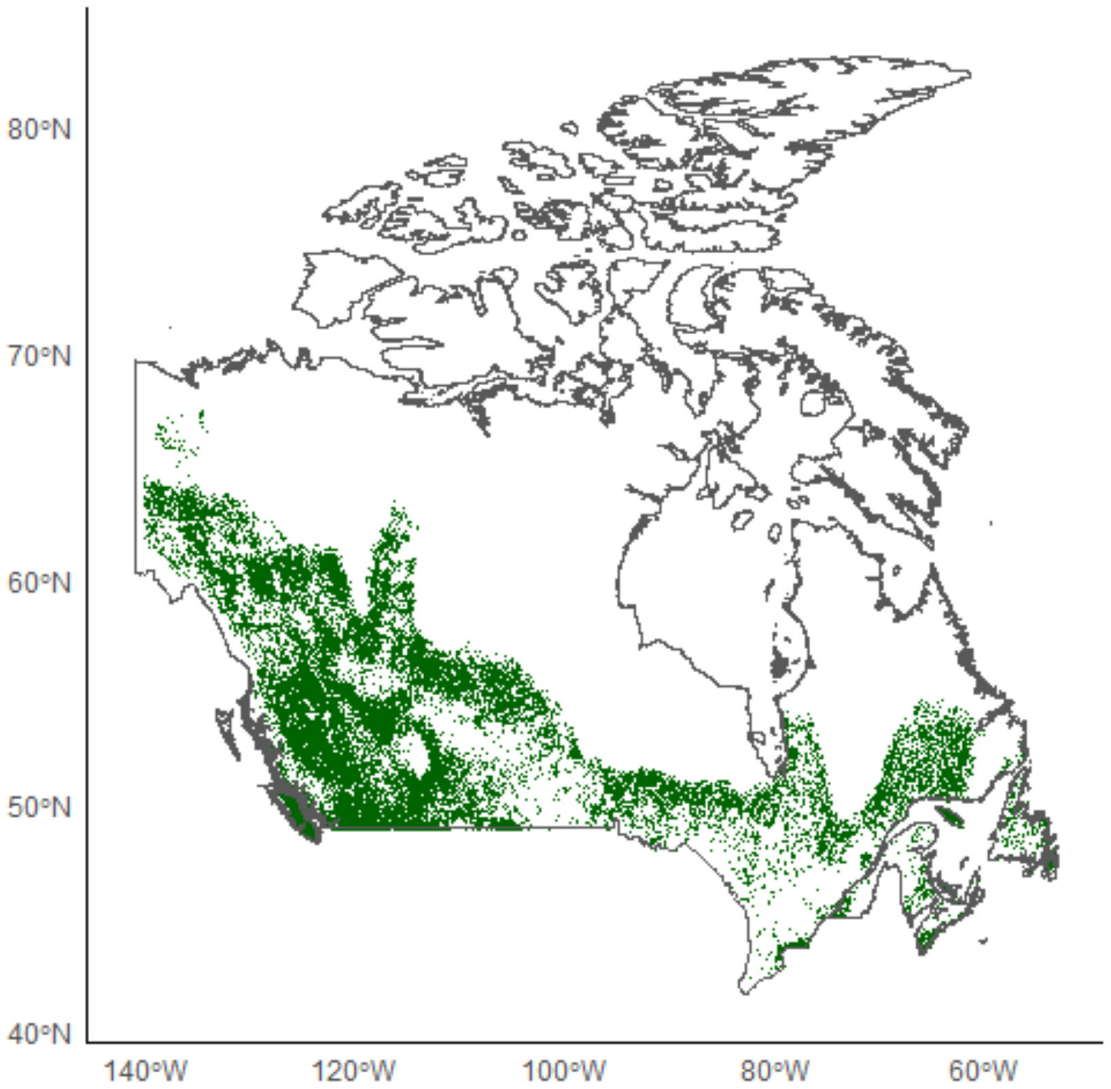
Canadian cougar binary habitat suitability map showing predicted presence at a sensitivity threshold of 0.9.

**FIGURE A2:**
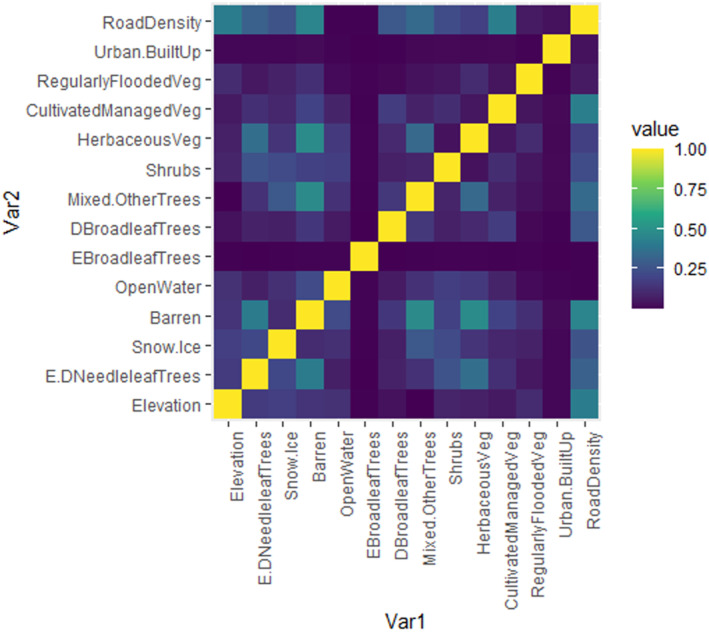
Pearson correlation heatmap between model variables.

**FIGURE A3:**
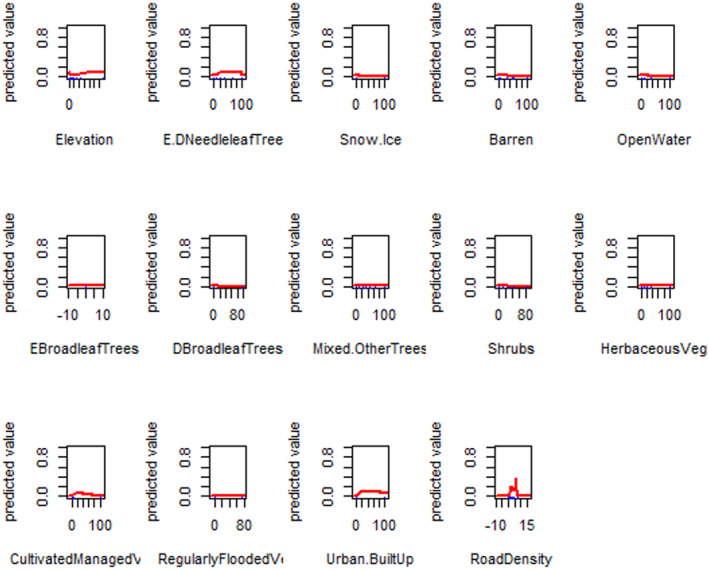
Response curves of model variables.

We obtained a total of 88 cougar occurrence points for use in the model after removal of duplicate occurrences. All the occurrence points were located in central to west Canada (Figure 1). As the Pearson correlation coefficient did not return any significant collinearity between habitat variables (Figure A2), all variables were included in the models. The validated model yielded an AUC of 0.94. High suitability was predicted throughout western Canada, particularly British Columbia, western Alberta, and north into Yukon (Figure 1). Habitat became less suitable moving east and north in much of Saskatchewan and through northern Ontario and Quebec (Figure 1). However, a band of suitable habitat ran across northern Ontario, up the eastern side of James Bay, and back down to a large area of suitable habitat in far eastern Quebec and southern Labrador (Figure 2). Road Density was the most important habitat variable, followed by Barren, Elevation, and Evergreen and Deciduous Needleleaf Trees (Table 2). The suitability of cougar habitat increased with increasing evergreen and deciduous needle leaf tree cover and elevation, limited cultivated managed vegetation and urbanization, and medium road density (Figure A3).

**TABLE 2:** Permutation importance of model variables.

Variable	Permutation importance
RoadDensity	56.7
Barren	12.7
Elevation	6.9
E.DNeedleleafTrees	6.6
Shrubs	4.5
Snow.Ice	3.6
RegularlyFloodedVeg	2.6
CultivatedManagedVeg	2.4
Urban.BuiltUp	1.7
DBroadleafTrees	1.4
Mixed.OtherTrees	0.8
HerbaceousVeg	0.1
OpenWater	0
EBroadleafTrees	0

**FIGURE 3:**
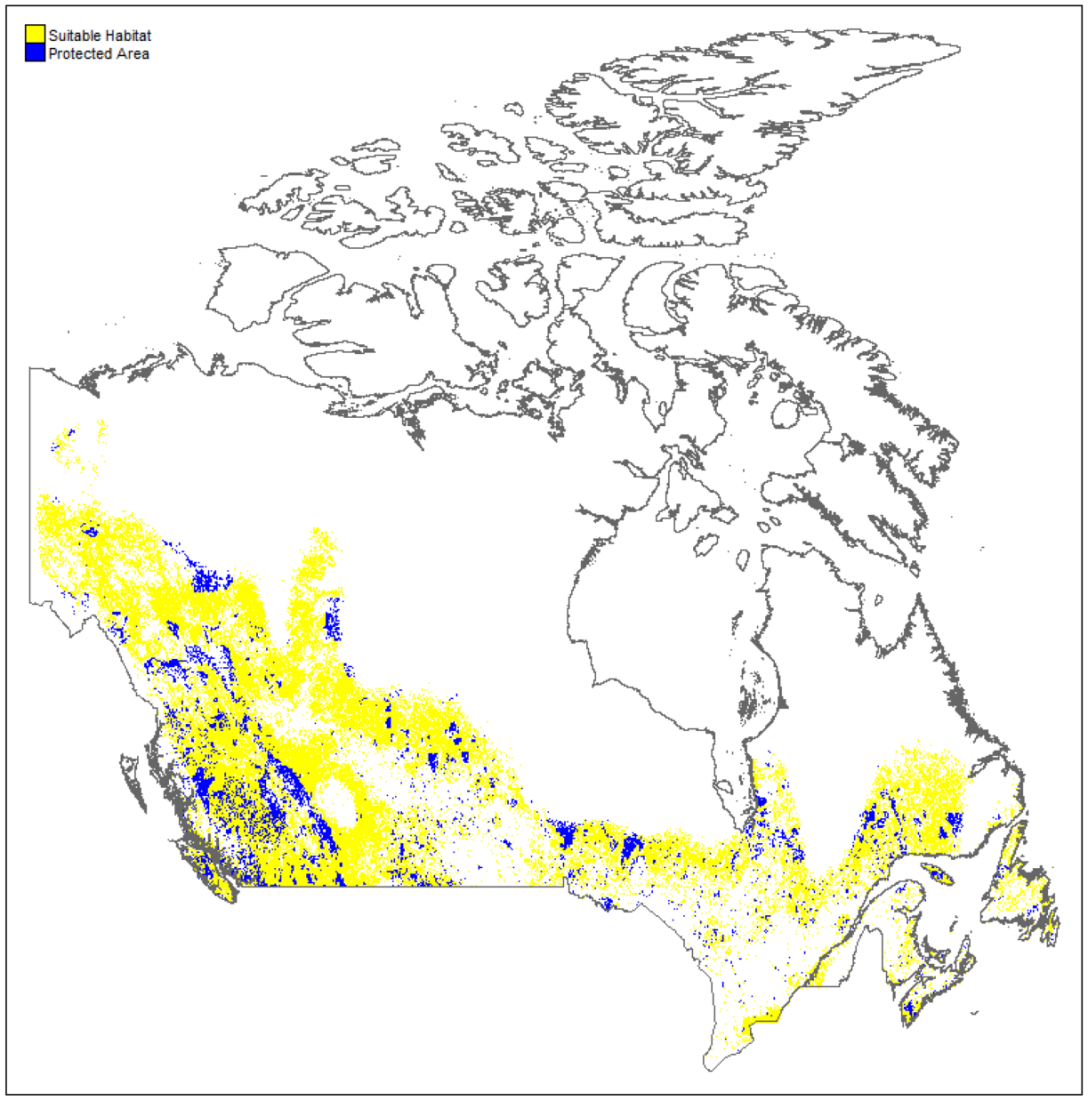
Binary habitat suitability map with overlapping Canadian Protected Areas and Other effective area‐based conservation measures shown in blue.

Protected and conserved areas of Canada highlight a lack of protection and connectivity across the country and little protected or conserved land through Manitoba, Ontario, and much of Quebec where the HSM indicates high habitat suitability for cougars (Figure 3). The proportion of protected area and OECM coverage of suitable habitat was 0.162. When only the protected area and OECM coverage larger than a cougar's approximately 300 km^2^ home range was considered, this proportion dropped to 0.134.

In the context of an eastward range expansion and potential likelihood for human–cougar encounters, the heavily populated corridor from the southern tip of Ontario in southeast Canada and east through Toronto, Ottawa, Montreal, and Quebec City is reflected in the absence of suitable habitat in the south‐east of the country (Figure 3).

## 4 DISCUSSION

### 4.1 Potential for cougar expansion

The HSM predicted suitable habitat for the potential future establishment of cougar populations in the central and eastern provinces. The importance of evergreen and deciduous needleleaf trees and, conversely, barren land is consistent with cougars' preference for forest cover (Gantchoff et al., 2021), with forest floor structure also providing concealment and protective microhabitats for den selection (Elbroch et al., 2015). Cultivated managed vegetation, which includes open agricultural land, increases habitat heterogeneity, which can support higher numbers of prey species (Prude & Cain, 2021).

Human impact from urbanization, agriculture, and energy development can greatly affect cougar habitat fragmentation and quality (Craighead et al., 2022; Holbrook et al., 2012). Roads in particular can have a detrimental impact on habitat suitability and connectivity (Dellinger et al., 2020; Mbuh & Vruno, 2018). Road density explained approximately 57% of modeled suitable habitat in this study, with suitability peaking at mid levels of road density. The importance of roads for predicting suitable cougar habitat could reflect a preference in large carnivores for using roads for movement and dispersal (Kautz et al., 2021). Transient cougars disperse at night, allowing them to move quickly through less suitable habitat and cross roads when vehicular traffic is quieter (Morrison et al., 2015) and avoiding roads by up to 400 m during daylight (Banfield et al., 2020). However, dispersing individuals face a high risk of mortality (Karelus et al., 2021), with anthropogenic features, including roads, and large tracts of open land typically being barriers to movement (Morrison et al., 2015), as reflected here where suitable habitat decreased with high road density. This means effective management requires consideration of vehicular traffic (Banfield et al., 2020). For example, in Banff National Park, Alberta, wildlife crossing structures have mitigated cougar vehicular mortality and restored habitat connectivity (Gloyne & Clevenger, 2001). Since cougars use wildlife crossing structures nearest to high quality habitat (Gloyne & Clevenger, 2001), provisions for suitable crossing structures that consider topography and other barriers to visibility around roadways can offset the avoidance of roads by cougars and reduce the likelihood for cougar–vehicle collisions (Banfield et al., 2020).

The presence of cougars on Vancouver Island and near the city of Vancouver in British Columbia could have contributed to the Urban/BuiltUp variable increasing habitat suitability. The higher predicted presence at a middle road density could be due to cougars using roads as movement corridors (Kautz et al., 2021). Both predicted uses of anthropogenic features could be due to ‘human observance’ and ‘material sample’ records being easier and more likely to occur as opportunistic public reports in readily human‐accessible areas (Gantchoff, Conlee, & Belant, 2022). Despite potential limitations of using GBIF public report data, these should not affect the determination of environmental variable importance or the general suitability of a large‐scale HSM (Gantchoff, Conlee, & Belant, 2022) such as ours.

Given their behavioral plasticity (Elbroch et al., 2013; Soria‐Díaz et al., 2018), cougars can adapt to variations in land cover (Avila‐Najera et al., 2017) and modify their prey‐hunting strategies (Elbroch et al., 2013; Prude & Cain, 2021), potentially enabling their eastward expansion through the fragmented suitable habitat east of mid‐Saskatchewan.

While climate was not included in this HSM, the impacts of climate change on cougar distribution are unknown (Avila‐Najera et al., 2017) and particular attention should be paid to potential impacts on preferred cougar habitats. Species' habitat changes due to climate change or anthropogenic activity as well as atypical movement behavior of individuals or populations can reduce model accuracy (Zurell et al., 2021). It is also possible that, since the predicted habitat is based on the recorded occurrence of established populations, individuals could roam outside predicted areas, especially in the case of generalist species (Gantchoff et al., 2021) or dispersing or traveling individuals and those whose home ranges contain unsuitable land; LaRue and Nielsen (2008, 2015) have modeled cougar dispersal across large patches of seemingly unsuitable habitat in the American Midwest.

### 4.2 Protected area coverage for expanding populations

Conservation implications of an eastward range expansion of cougars in Canada include managing and protecting land from human development and impassable roadways, particularly for supporting cougar reproductive behaviors. For example, communication and female denning behaviors require a buffer area around development four times larger than that required for non‐reproductive movement and feeding behaviors (Wilmers et al., 2013). Therefore, the availability of sufficiently large and connected habitat patches is paramount for maintaining viable cougar populations (Dellinger et al., 2020; Gantchoff et al., 2021; Holbrook et al., 2012).

Resident cougars generally require sufficient suitable habitat for a home range size of approximately 300 km^2^ (Riley et al., 2021); however, smaller patches can be equally important for dispersing individuals and maintaining connectivity between reproductive populations (Dickson et al., 2013). The majority of the protected area and OECM coverage of suitable habitat is in the far western provinces. This highlights the importance of maintaining large protected areas in the east, such as the 29,000 km^2^ Pimachiowin Aki protected area straddling the border of Manitoba and Ontario (UNEP‐WCMC & IUCN, 2022), more than 95 times the area required for resident cougars. Other notable areas of overlap between suitable habitat and formal protected areas are Wabakimi Provincial Park (8929 km^2^, UNEP‐WCMC & IUCN, 2022) in Ontario and the Caribous‐Forestiers‐de‐Manouane‐Manicouagan Territorial reserve (2380 km^2^, UNEP‐WCMC and IUCN, 2022) and the 4089 km^2^ Proposed vallée de la rivière Natashquan Biodiversity Reserve in Quebec (UNEP‐WCMC & IUCN, 2022). The majority of other overlapping areas are significantly smaller (approximately 500 km^2^, UNEP‐WCMC & IUCN, 2022) and, therefore, unlikely to support more than one reproductive male within the protected area alone. Given that formal protected areas are unlikely to effectively conserve expanding cougar populations, and OECMs are currently lacking in Manitoba, Ontario, and Quebec (Government of Canada, 2024), private land will be important for managing both suitable habitat and connectivity (Karelus et al., 2021), particularly in the case of large commercial forestry operations (Gantchoff et al., 2021). Future research could also attempt to determine the carrying capacity of different areas and perform connectivity analyses between protected areas.

### 4.3 Human–wildlife conflict with an expanding population

The patterns and speed with which cougars expand their range boundaries are relatively poorly understood. Males disperse substantially further than females, with dispersing subadult males often traveling 400 km or more before establishing a home range (LaRue & Nielsen, 2008) and are most often recorded as those found beyond established range boundaries (Morrison et al., 2015). Dispersing subadult males have been observed establishing isolated home ranges in fragmented habitat beyond the limits of their existing range boundaries (Riley et al., 2021), reflecting the adaptable nature of cougars (Prude & Cain, 2021; Soria‐Díaz et al., 2018). Yet establishing a reproductive population east of existing range boundaries in Canada is dependent on female dispersal (Gantchoff et al., 2021; LaRue & Nielsen, 2015). Since dispersing cougars can remain in a temporary home range for up to 1 year (Karelus et al., 2021), long‐term monitoring is required to determine permanent population expansion.

Land protection alone is insufficient for protecting cougar populations (Vickers et al., 2015). As cougars enter new territory, understanding the perception and likelihood of cougar–human encounters is key to their successful conservation and the safety of livestock and humans (Adams Knopff et al., 2016; Gantchoff et al., 2021). The survival of isolated populations is limited by vehicular collisions and livestock depredation retaliation (Vickers et al., 2015), while changes to the age and social structure of populations due to trophy hunting (Teichman et al., 2016) and shifting diets from native to domestic and invasive species (Moss et al., 2016) potentially increase the likelihood of human–cougar conflict. Although cougars are perceived by many to be fearsome animals (Campbell, 2013) in the western provinces of British Columbia and Alberta, a majority of residents valued cougars for increasing quality of life and serving a useful ecological function (Adams Knopff et al., 2016; Campbell, 2013). However, this value is placed on cougars residing in the wilderness, away from peoples' homes (Adams Knopff et al., 2016). Given that dispersing, transient cougars are likely to be in proximity to anthropogenic development (Karelus et al., 2021; Morrison et al., 2015; Ramírez‐Álvarez et al., 2021), cougars observed close to residential areas are unlikely to permanently remain in the area. Indeed, that habitat suitability increased with elevation may be indicative of cougars' avoidance of human activity, especially as occurrence records came from western provinces that are highly populated. That the predicted suitable habitat in the central and eastern provinces does not coincide with existing highly human‐populated areas means that proactive and informed management plans enacted in advance of cougar population establishment could reduce the likelihood of the current human–wildlife management challenges faced in the west.

The eastward movement of Canadian cougars is most likely to be hindered by misguided or insufficient conservation and land management. Since cougars can adapt to regular human disturbance (Banfield et al., 2020; Buderman et al., 2018; Morrison et al., 2014) and urban landscapes (Moss et al., 2016; Riley et al., 2021), careful future development in exurban areas is necessary to reduce potential conflict with cougar populations as they increase alongside increasing anthropogenic development (Adams Knopff et al., 2016). This is particularly important in areas predicted to be highly suitable cougar habitat in the central and eastern provinces of Canada, since without appropriate management and mitigation these intermediate‐ and low‐density suburban and exurban communities have the potential to become ecological traps and sources of human–cougar conflict due to altered predator–prey relationships (Moss et al., 2016; Nisi et al., 2022). Here, modeling human activity together with temporal changes in cougar behavior (Curras et al., 2022; Morrison et al., 2014) would provide insight for future developments, since human activity appears to deter cougars more than the presence of human physical infrastructure alone (Morrison et al., 2014; Suraci et al., 2019).

## 5 CONCLUSION

The HSM suggests there is sufficient suitable habitat for an eastward expansion of cougar populations in Canada. Behavioral plasticity may allow cougars to move through areas of unsuitable habitat, including urban areas, but where areas of high road density intersect highly suitable cougar habitat, protected areas provide important levels of coverage. Proactive, informed management will be required for the establishment, support, and maintenance of expanding populations in the more fragmented habitat in the central and eastern provinces. These eastern areas can draw on the experiences of the western provinces to adapt and develop locally relevant conservation actions to protect cougar populations and minimize human–cougar conflict. Such actions include maintaining habitat connectivity; wildlife crossing structures; education of landowners and the public; urban planning and livestock husbandry practices; and adaptive hunting management. This study demonstrates the value of modeling habitat suitability to gain insight into expanding large carnivore populations.

## AUTHOR CONTRIBUTIONS

**Jennifer A. Christoff:** Conceptualization (lead); data curation (lead); formal analysis (lead); methodology (equal); visualization (equal); writing – original draft (lead); writing – review and editing (equal). **Eleanor S. Devenish‐Nelson:** Methodology (equal); supervision (lead); visualization (equal); writing – review and editing (equal).

## CONFLICT OF INTEREST STATEMENT

The authors have no conflicts of interest to declare.

## Supporting information


Appendix S1.

## Data Availability

The data that support the findings of this study are available at https://zenodo.org/record/7339829. These data were derived from the following resources available in the public domain: Global Biodiversity Information Facility (https://www.gbif.org).
