# Supplementary material for: Habitat suitability and protected area coverage for an expanding cougar Puma concolor population in Canada
Source: Ecol Evol. 2024 Aug 30;14(9):e70228. doi: 10.1002/ece3.70228 (PMC11364782; doi:10.1002/ece3.70228)
Supplement: Supplementary file 1 — Appendix S1. [file ECE3-14-e70228-s001.docx]

**Appendix B – R Code**

# INSTALL AND LOAD PACKAGES #######################################

# Install and load tidyverse

install.packages("tidyverse")

library(tidyverse)

# Install and load sp

install.packages("sp")

library(sp)

# Install and load raster

install.packages("raster")

library(raster)

# Install and load rJava

install.packages("rJava")

library(rJava)

# Install and load rgbif

install.packages("rgbif")

library(rgbif)

# Install and load rgdal

install.packages("rgdal")

library(rgdal)

# Install and load dismo

install.packages("dismo")

library(dismo)

# IMPORT SPECIES DATA ##############################################

# Import Puma concolor data from GBIF

key <- name_suggest(q='Puma concolor', rank='species')$data$key[1]

PconcolorRAW <- occ_search(taxonKey=key, country='CA', hasCoordinate=T, hasGeospatialIssue=F, basisOfRecord='HUMAN_OBSERVATION;MATERIAL_SAMPLE')

as.data.frame(PconcolorRAW$data)

Pconcolorpts <- cbind.data.frame(PconcolorRAW$data$decimalLongitude,PconcolorRAW$data$decimalLatitude)

# Clean P concolor point data

duplicated(Pconcolorpts)

Pconcolorocc<-distinct(Pconcolorpts)

# Use Zenodo file

Pconcolorocc<-read.csv('data/cleaned_data_for_zenodo.tsv', sep='\t')

# IMPORT HABITAT DATA ##############################################

# Obtain Canadian and Provincial boundaries

CAN<-getData('GADM', country='CAN', level=0)

PROV<-getData('GADM', country='CAN', level=1)

CENSUS<-getData('GADM', country='CAN', level=2)

Alt<-getData('alt', country='CAN', mask=T)

# Import and crop elevation data

ElevRAW <- raster('data/elevation.tif')

crop<-c(-141.1, -52.6, 41.6, 83.2)

ElevCROP <- crop(ElevRAW, crop)

Elevation <- mask(ElevCROP, Alt)

ext<-extent(Alt)

# Import, stack and crop land use data

landusefiles <- list.files(path='data/landuse', full.names=T)

LandUseStack <- stack(landusefiles)

LandUseCROP<-crop(LandUseStack, crop)

LandUse<-mask(LandUseCROP, Alt)

# Import and crop road density data

rddens <- raster('data/roaddensity/GRIP_RdDens.tif')

rddenscrop <- crop(rddens, crop)

roaddensity <- resample(rddenscrop, Elevation, method='ngb', filename='data/roaddensity/roaddensity')

roaddensity <- mask(rddenscrop, Alt)

roaddensity <- raster('data/roaddensity/roaddensity.grd')

# PLOT HABITAT AND P CONCOLOR DATA ################################

# Plot habitat data

#plot(Elevation)

#plot(CAN, add=T)

#points(Pconcolorocc, col='blue', pch=18, cex=0.9)

#plot(LandUse)

# Plot P concolor data

#plot(CAN)

#points(Pconcolorocc, col='blue', pch=18, cex=0.9)

# SET UP THE MODEL #################################################

# Set Predictor Variables

PredVar<-stack(Elevation, LandUse, roaddensity)

names(PredVar)<-c("Elevation", "E/DNeedleleafTrees", "Snow/Ice", "Barren", "OpenWater", "EBroadleafTrees", "DBroadleafTrees", "Mixed/OtherTrees", "Shrubs", "HerbaceousVeg", "CultivatedManagedVeg", "RegularlyFloodedVeg", "Urban/BuiltUp", "RoadDensity")

# Set training and testing points

bg<-randomPoints(PredVar, n=5000, p=Pconcolorocc, ext=ext)

colnames(bg)<-c('lon','lat')

# For presence data

group<-kfold(Pconcolorocc, 5)

pres_train<-Pconcolorocc[group != 1, ]

pres_test<-Pconcolorocc[group == 1, ]

# For background data

group<-kfold(bg, 5)

bg_train<-bg[group != 1, ]

bg_test<-bg[group == 1, ]

# Check data

plot(CAN)

points(pres_test, col = "purple", pch = 20, cex = 0.9)

points(bg_test, col = "turquoise", pch = 20, cex = 0.9)

legend("topright", legend = c("Testing presence", "Testing background"), cex = 0.7,

pt.cex = 1, pch = 20, col = c("purple", "turquoise"), bty = "n")

# RUN THE MODEL ##################################################

# Train Maxent model

xm<-maxent(PredVar, pres_train, a = bg_train, removeDuplicates = T, args = c("jackknife=true"))

# Train with all data

xm<-maxent(PredVar, Pconcolorocc, a = bg, removeDuplicates = T, args = c("jackknife=true"))

# Run model

model <- predict(xm, PredVar, ext = ext, filename = "maxentalldata3.asc", progress = "text",

overwrite = T)

# Assign geographic projection

proj4string(model) <- CRS("+proj=longlat +datum=WGS84 +ellps=WGS84 +towgs84=0,0,0")

writeRaster(model, filename="predictalldata3.tif", bylayer=T, overwrite=T)

# Test for collinearity

install.packages("ellipse")

library(ellipse)

install.packages("ENMTools")

library(ENMTools)

install.extras()

raster.cor.plot(PredVar, method="pearson")

#install.packages("sdmpredictors")

#library(sdmpredictors)

#PCMatrix<-pearson_correlation_matrix(PredVar)

#write.csv(PCMatrix, "PCorrMatrix.csv")

#jnk=layerStats(PredVar, 'pearson', na.rm=T)

#cm=jnk$'pearson correlation coefficient'

#plotcorr(PCMatrix, col=ifelse(abs(PCMatrix)>0.7, "red", "black"), mar=c(0,0,0,0))

#plotcorr(PCMatrix, col=ifelse(abs(PCMatrix)>0.7, "red", "black"), mar=c(0,0,0,0), numbers=T)

#plotcorr(PCMatrix, col=ifelse(abs(PCMatrix)>0.7, "red", "black"), mar=c(0,0,0,0), numbers=T, type="upper")

# Evaluate AUC

eval <- evaluate(pres_test, bg_test, xm, PredVar)

AUC <- eval@auc

# Evaluate AUC with all data

eval <- evaluate(Pconcolorocc, bg, xm, PredVar)

AUC <- eval@auc

# Threshold probability

tr <- threshold(eval, stat = "sensitivity", sensitivity = 0.9)

# Area above threshold

hr <- calc(model, fun = function(x) {ifelse(x > tr, 1, NA)})

# Plot contribution of predictor variables

plot(xm, cex = 1, bg = "black")

response(xm)

# Determine permutation importance (Maxent HTML site)

xm

# Plot AUC-ROC

plot(eval, "ROC")

# Map predicted habitat

plot(model)

points(pres_train, col = "blue", pch = 20, cex = 0.9)

points(pres_test, col = "red", pch = 20, cex = 0.9)

# With all data

points(Pconcolorocc, col = "blue", pch = 20, cex = 0.9)

# Add outline of Canada

plot(CAN, add = T)

legend("topright", legend = c("ActualPresenceTrain", "ActualPresenceTest"), cex = 0.7, pt.cex = 1, pch = 20,

col = c("blue", "red"), bty = "n")

# With all data

legend("topright", legend = c("ActualPresence"), cex = 0.7, pt.cex = 1, pch = 20,

col = c("blue"), bty = "n")

writeRaster(hr, filename="binaryalldata3.tif", bylayer=T, overwrite=T)

# Plot mean predicted habitat

plot(hr, legend = F)

points(Pconcolorocc, col = "blue", pch = 20, cex = 0.9)

plot(CAN, add = T)

legend("topright", legend = c("PredictedPres>Threshold", "ActualPresence"), cex = 0.7,

pt.cex = 1, pch = 20, col = c("yellow", "blue"), bty = "n")

# Protected Areas map

# The input file geodatabase

require(rgdal)

fgdb <-("data/WDPA_WDOECM_Mar2022_Public_CAN.gdb")

# List all feature classes in a file geodatabase

subset(ogrDrivers(), grepl("WDPA_WDOECM_Mar2022_Public_CAN.gdb", name))

fc_list <- ogrListLayers(fgdb)

print(fc_list)

# Read the feature class

fc <- readOGR(dsn=fgdb, layer='WDPA_WDOECM_poly_Mar2022_CAN')

# Rasterize and plot

proj4string(PAmap) <- CRS("+proj=longlat +datum=WGS84 +ellps=WGS84 +towgs84=0,0,0")

install.packages("terra")

library(terra)

PAmap<-rasterize(fc, Alt, mask=TRUE)

plot(hr, legend=F)

points(Pconcolorocc, col='blue', pch=18, cex=0.9)

plot(PAmap, col='green', border=NA, legend=F, add=T)

plot(CAN, add=T)

legend("topright", legend = c("PredictedPres>Threshold", "ActualPresence", "ProtectedAreas"), cex = 0.7,

pt.cex = 1, pch = 20, col = c("yellow", "blue", "green"), bty = "n")

# PLOT HABITAT SUITABILITY MAP ##################################

rm(list=ls()) ### Clears the working environment

library(terra)

library(sf)

library(sp)

library(tmap)

library(geodata)

library(ggplot2)

library(viridis)

library(tidyterra)

### Define working directory for saving

wd <- getwd()

### Read in layers ####

### Download Canada outline (or read in below)

#CAN <- gadm(country="CAN", level=0, path=wd)

# <- st_as_sf(gadm(country="CAN", level=0, path=wd))

## save layer

#st_write(st_as_sf(CAN), dsn=wd, layer = "CAN.shp", driver = "ESRI Shapefile")

### Read in layer

CAN <- read_sf("CAN.shp")

### Read in prediction raster

pred <- rast("predictalldata3.tif")

plot(pred)

plot(CAN, add=T)

### Read in binary prediction raster

#### Convert raster values to factors (otherwise it’s plotting as continuous values which messes up the colour fill)

binpred <- as.factor(rast("binaryalldata3.tif"))

plot(binpred,col="darkgreen")

plot(CAN, add=T)

### Cougar occurrence points from Zenodo data

points <- read.csv("data/cleaned_data_for_zenodo.tsv",header=T, sep='\t')

### Convert points to spatial

pointssp <- st_as_sf(points,

coords=c("PconcolorRAW.data.decimalLongitude","PconcolorRAW.data.decimalLatitude"),

crs=4326)

### Plotting ###

### Plot the full prediction - can change multiple settings (See below)

p1 <- ggplot() +

geom_spatraster(data = pred) +

scale_fill_viridis(na.value="white") +

geom_sf(data=pointssp,colour="orange",size=2)

p1

tiff(filename="CougarSuitableHabitatalldata3.tif",width=800,height=800,

pointsize=20,

units="px",compression="lzw")

p1

dev.off()

## may have to change default graphics to 'AGG' in RStudio Global Option to get degrees to show properly

p2 <- ggplot() +

geom_spatraster(data = binpred)+#,show.legend = FALSE) +

geom_spatvector(data=CAN,fill="transparent") + ### set this to be transparent

scale_fill_manual(values="darkgreen",na.value = "transparent") +

theme_minimal(base_family = "sans serif") +

theme(

plot.background = element_rect(fill = "white", color = "white"),

panel.grid.major = element_blank(),

axis.line = element_line(colour = "black"),

axis.title = element_text(size = 20),

legend.position="none"

)

p2

# DETERMINE AND PLOT PROTECTED AREA OVERLAP ####################

rm(list=ls())

library(terra)

library(sf)

library(tidyverse)

library(geodata)

sf_use_s2(FALSE)

wd <- getwd()

### Read in layers #####

## Read in binary prediction

binpred <- rast("binaryalldata3.tif")

#### Coverage by PAs ####

## Read in dissolved Protected Areas shapefile (doesn't contain individual PA info)

pa_dis <- read_sf("CPCAD_Canada_Dissolved.shp")

#### Check it's in the same coordinates as raster

pa_dis = st_transform(pa_dis, crs(binpred))

### Check and fix geometry (sometimes needs fixing after transforming)

pa_dis <- st_make_valid(pa_dis)

### Crop the binary prediction to the PAs shapefile ####

### this can take a while - so read in the saved version below

#bin_pacrop <- mask(binpred,pa_dis)

### Save raster output

#writeRaster(bin_pacrop, "CougarBinary_PAs.tif", overwrite=T)

## once it’s saved it can be read in next time instead of cropping

bin_pacrop <- rast("CougarBinary_PAs.tif")

### Download Canada outline (or read in below)

#CAN <- gadm(country="CAN", level=0, path=wd)

# <- st_as_sf(gadm(country="CAN", level=0, path=wd))

## Save layer

#st_write(st_as_sf(CAN), dsn=wd, layer = "CAN.shp", driver = "ESRI Shapefile", append=F)

### If it’s already saved it doesn't need to be downloaded each time

CAN <- read_sf("CAN.shp")

## Plot just to check layers ###

plot(binpred, legend=F)

plot(bin_pacrop, col="red",add=T, legend=F)

plot(CAN, add=T)

## can also add PAs on top - this takes a long time

#plot(pa_dis, add=T)

#plot(CAN, add=T)

### Make Habitat and PA combined map ###

install.packages('tmap')

library(tmap)

tm_shape(binpred) + tm_raster(legend.show=F, palette="yellow", title="Suitable Habitat")+

tm_shape(bin_pacrop) + tm_raster(legend.show=F, palette="blue", title="Protected Areas")+

tm_shape(CAN)+

tm_borders()+

tm_add_legend(type = "fill",

labels = c("Suitable Habitat","Protected Area"),#NULL,

col = c("yellow","blue"),

border.lwd = 0.5)

### Proportion PA coverage #####

### This works out proportion of suitable habitat with PAs

pa_sum <- global(bin_pacrop==1, sum, na.rm=TRUE) ### PA area == 1

bin_sum <- global(binpred==1, sum, na.rm=TRUE) ### total area == 1

prop.pa <- pa_sum/bin_sum ### Proportion of total suitable area within PAs

prop.pa

### Proportion of PAs with area > cougar home range #####

## Read in Protected Areas shapefile

pa_all <- read_sf("CPCAD_Canada_Cleaned_duplicates 1.shp")

#### Check it's in the same coordinates as raster

pa_all = st_transform(pa_all, crs(binpred))

### Check and fix geometry (sometimes needs fixing after transforming)

pa_all <- st_make_valid(pa_all)

### Define home range size (300 km2)

home_range <- 300

### Remove PAs area > home range - using the area in km column in shapefile

#pa_all$area_sf_km <- st_area(pa_all)/100000 ### calculates area in m as this is the unit of this polygon - possible to change this but can also just convert to km

#pa_all_home_range <- pa_all[pa_all$Area_km > home_range,]

### Dissolve polygon to make it easier to work with

## this step not strictly necessary as takes time but can make it quicker to run analyses

#pa_all_home_range_diss <- pa_all_home_range %>%

# summarize(geometry = st_union(geometry))

### Save polygon output

#st_write(pa_all_home_range, "PAs_Smaller_HomeRange.shp",append=T)

#### Crop the binary prediction to the PAs/home range shapefile ####

### this can take a while - so read in the saved version below

#bin_pa_range_crop <- mask(binpred,pa_all_home_range_diss)

## save raster output

#writeRaster(bin_pa_range_crop, "CougarBinary_PA_HomeRange.tif",overwrite=T)

## once we've saved it we can read it in next time instead of cropping

#bin_pa_range_crop <- rast("CougarBinary_PA_HomeRange.tif")

## Plot just to check it's removed small PAs ###

#plot(bin_pacrop, legend=F)

#plot(bin_pa_range_crop, col="red",add=T)

#plot(CAN, add=T)

### Proportion PA > home range coverage #####

### this works out proportion of suitable habitat with PAs > average home range size

bin_hr_sum <- global(bin_pa_range_crop==1, sum, na.rm=TRUE) ### PA area == 1

prop_pa_hr <- bin_hr_sum/bin_sum ### Proportion of total suitable area within PAs

prop_pa_hr
